# Rheological and Performance Properties of a Bituminous Binder Modified with Date Kernel Powder

**DOI:** 10.3390/ma19061120

**Published:** 2026-03-13

**Authors:** Ceren Beyza İnce

**Affiliations:** Department of Civil Engineering, Faculty of Engineering and Natural Sciences, Malatya Turgut Ozal University, Malatya 44900, Turkey; ceren.ince@ozal.edu.tr; Tel.: +90-05078315591

**Keywords:** bituminous binder, agricultural residue, modification, high temperature performance, rheology, sustainable pavement

## Abstract

**Highlights:**

- 15% DKP significantly improves high-temperature rutting resistance.
- DKP-modified binders enhance low-temperature cracking resistance.
- Fatigue life at intermediate temperatures increases with DKP addition.
- DKP offers a sustainable, low-cost alternative for bitumen modification.
- Valorization of date kernel waste supports environmentally friendly pavements.

**Abstract:**

This study presents an experimental investigation into the direct use of date kernel powder (DKP) as a biomass-based modifier for bituminous binders, with the aim of evaluating its feasibility as a sustainable binder modifier. DKP was incorporated into a conventional bituminous binder at different contents (5, 10, 15, and 20 wt.% by weight of binder), and its physicochemical properties were characterized using SEM, XRD, and FTIR. The rheological and performance properties of the modified binders were evaluated through conventional tests, aging procedures, rotational viscosity (RV), dynamic shear rheometer (DSR), bending beam rheometer (BBR), and linear amplitude sweep (LAS) testing, and the performance grades (PG) of all binders were determined. The results indicate that DKP addition increases binder stiffness and reduces temperature susceptibility while maintaining acceptable fatigue and low-temperature performance. Performance grading results showed that the high-temperature grade increased from PG 64 to PG 70 and the low-temperature grade improved from PG-22 to PG-34 at a DKP content of 15%. LAS test results indicated that fatigue life was maintained or improved at intermediate temperatures. Among the tested contents, 15% DKP provided the most balanced performance considering performance grade improvement, fatigue behavior, and workability characteristics, while higher contents resulted in increased stiffness. Overall, the findings suggest that DKP is a promising modifier for bituminous binders at the binder level. However, further studies at the mixture and field scale are recommended to confirm the long-term engineering applicability of DKP-modified binders.

## 1. Introduction

In highway engineering, durability and sustainability are critical factors governing the service life of pavements. In recent years, rapid population growth and the corresponding increase in transportation demand have subjected road pavements to heavier traffic loads and more variable environmental conditions. As a result, premature pavement deterioration has become more frequent, leading to increased maintenance and rehabilitation requirements. Accordingly, improving the performance of asphalt pavements and extending their service life remain among the primary research priorities in road engineering [1,2].

Asphalt pavements are mainly composed of aggregates and bituminous binder. Aggregates constitute the majority of the asphalt mixture volume and largely determine load-bearing capacity, resistance to permanent deformation, skid resistance, and overall mechanical strength. Bituminous binder acts as the binding material that holds aggregate particles together and plays a decisive role in governing the flexible behavior of pavements, moisture resistance, and performance under repeated traffic loading [2,3].

Owing to its viscoelastic nature, bituminous binder contributes to stress redistribution within the pavement structure, thereby enhancing resistance to fatigue cracking, permanent deformation, and thermal distress. The rheological behavior of bituminous binder, particularly under cyclic loading and temperature variations, is one of the key parameters influencing pavement longevity. Therefore, an in-depth understanding of bituminous binder rheology is essential for accurately evaluating the long-term performance of asphalt pavements [4,5].

Despite these advantages, the viscoelastic nature of bituminous binder also makes it highly sensitive to temperature and loading conditions, which can result in rutting, thermal cracking, and fatigue-related damage [6]. To mitigate these drawbacks, numerous studies have focused on improving bituminous binder performance through modification using various additives. Conventional modifiers such as styrene–butadiene–styrene (SBS) [7,8], various polymers [9,10,11,12], fibers [13,14] and chemical additives [15,16,17] have been extensively investigated and shown to enhance pavement performance. However, high costs, supply limitations, and environmental concerns associated with these materials have increasingly directed research toward alternative and sustainable modifiers. In this regard, biomass-based agricultural by-products have attracted growing attention due to their environmental and economic advantages [1,2].

In the context of material utilization, the term “waste” generally refers to materials that have completed their service life and require disposal [18,19], whereas “by-products” denote materials generated during production processes that can be reused when appropriately evaluated [20]. Biomass-derived materials obtained from agricultural activities fall into the latter category and offer significant potential for value-added applications in engineering fields [21].

Among biomass-based materials, plastic-derived wastes have been widely investigated in bituminous binder modification, with numerous studies reporting favorable performance outcomes for materials such as polyethylene terephthalate (PET) bottles and plastic bags [22,23,24]. In addition, agricultural by-products originating from sunflower [1,25,26], olive [27,28,29], cotton [30,31] and similar crops have been successfully incorporated into bituminous binder and hot mix asphalt to improve performance characteristics. These materials are considered promising alternatives due to their low cost, wide availability, and environmentally friendly nature, while also contributing to waste/residue management mitigation and more efficient use of natural resources [32,33,34].

Date palm is a major agricultural product cultivated extensively in hot-climate regions, particularly in the Middle East and North Africa, with a high annual production volume. Global date production reaches several million tons per year, with a substantial share originating from countries such as the United Arab Emirates (approximately 1.5 million tons), Saudi Arabia, Egypt, Iran, and Iraq. Date seeds account for nearly 10% of the fruit mass; however, their utilization in the food industry remains limited. Consequently, these seeds are largely discarded or incinerated, with only a minor fraction used as animal feed. Furthermore, Saudi Arabia alone is reported to generate more than 200,000 tons of date kernel by-products annually. This situation leads to environmental concerns while simultaneously representing an underutilized biomass resource with significant valorization potential [21,35,36]. In date-producing regions such as the Middle East and North Africa, date kernels are generated in large quantities as biomass by-products of consumption and processing, making their collection feasible for engineering applications [37].

Although date kernels have been explored in a limited number of studies for applications such as energy production [37,38], activated carbon [39,40], and filler material [41,42], research focusing on the direct use of date kernel powder in bituminous binder modification remains scarce [21]. Existing studies predominantly convert date kernels into ash through combustion, whereas their direct utilization in powder form has not been sufficiently investigated [43,44,45]. Owing to its lignocellulosic structure, porous morphology, and polar functional groups, date kernel powder exhibits a strong potential for physical interaction with the bituminous binder matrix. Therefore, its application as a modifier in bituminous binders represents a promising research direction for both high-value utilization of agricultural by-products and the development of sustainable and cost-effective asphalt pavements [46].

Although several studies have investigated date kernel-derived materials or similar biomass additives in binder modification, important aspects remain insufficiently understood. In particular, the combined effects of DKP content on rutting resistance, fatigue performance, and low-temperature cracking behavior have not been systematically evaluated within a unified experimental framework. While previous studies on biomass-based modifiers generally reported increased stiffness and improved high-temperature performance, the balance between stiffness improvement and low-temperature or fatigue performance remains uncertain for DKP-modified binders. Furthermore, limited information is available regarding the aging sensitivity of DKP-modified binders and the relationships between microstructural characteristics and rheological performance. These uncertainties make it difficult to assess the engineering feasibility and performance balance of DKP-modified binders [43,44].

In this study, the effects of date kernel powder (DKP), an agricultural by-product, on the rheological and performance properties of bituminous binders were systematically investigated. Modified binders were produced by incorporating DKP at different contents, and their conventional properties, workability, and Superpave performance parameters were evaluated. In addition, fatigue behavior at intermediate temperatures was assessed using the linear amplitude sweep (LAS) test. The findings aim to contribute to the growing body of knowledge on biomass-based modifiers and to demonstrate the feasibility of directly using agricultural by-products in sustainable pavement engineering applications at the binder level.

In order to address the identified research gaps, the main objectives of this study are summarized as follows:

- To characterize the microstructural and chemical properties of date kernel powder using SEM, XRD, and FTIR analyses.
- To evaluate the effects of DKP content on the physical and workability properties of bituminous binders using conventional tests and rotational viscosity measurements.
- To investigate the rheological and performance behavior of DKP-modified binders at high and intermediate temperatures using DSR tests and performance grading.
- To assess the low-temperature performance and thermal cracking resistance of binders using BBR tests and PG classification.
- To evaluate fatigue performance and aging-related behavior of DKP-modified binders using LAS tests together with RTFO and PAV aging procedures.

Based on these objectives and the identified research gaps, this study was conducted under the following testable hypotheses:

(i) Increasing DKP content is expected to improve high-temperature rutting resistance due to increased binder stiffness and internal structural reinforcement.
(ii) DKP modification is expected to maintain or improve fatigue and low-temperature performance despite the increase in stiffness.
(iii) DKP-modified binders are expected to maintain acceptable rheological performance after short-term and long-term aging.
(iv) The microstructural and chemical characteristics of DKP are expected to support the observed rheological and performance behavior of the modified binders.

The main scientific contributions of this study can be summarized as follows:

- Direct utilization of date kernel powder as a binder modifier without combustion or chemical treatment.
- A comprehensive evaluation of DKP-modified binders including conventional, rheological, performance-based, and microstructural analyses.
- Integrated assessment of high-, intermediate-, and low-temperature performance through Superpave-based testing and performance grading.
- Evaluation of aging behavior of DKP-modified binders using RTFO and PAV procedures.
- Identification of an optimum DKP content based on the balance between performance improvement and workability.

## 2. Materials and Methods

**Figure 1:**
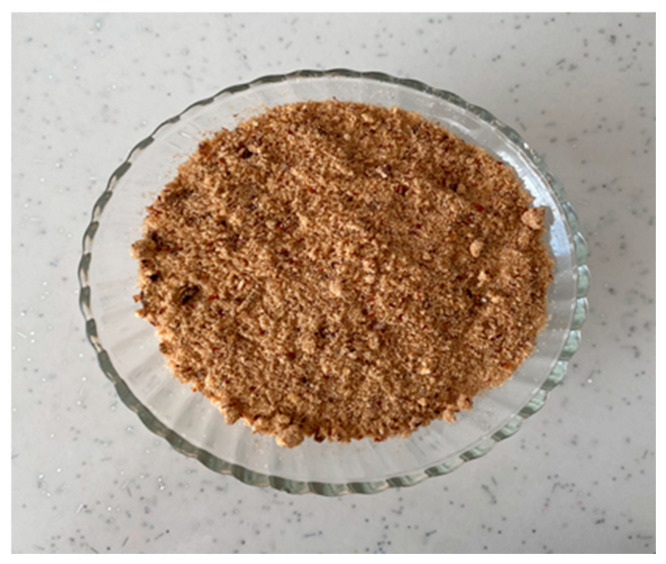
Date kernel powder (DKP).

In this study, B50/70 bituminous binder (Kırıkkale/TUPRAS refinery), which is used in high-temperature regions, was preferred as the binder. Date kernel powder (DKP) was used as the additive (Figure 1).

### Obtaining of DKP and Preparation of Modified Binders

**Figure 2:**
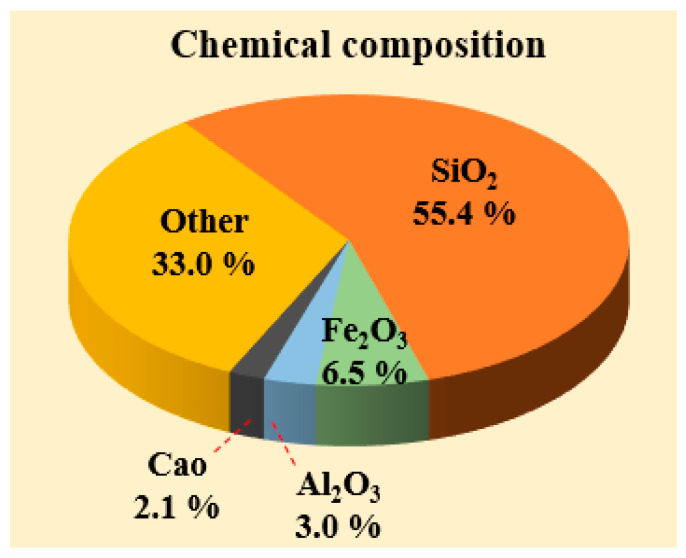
Chemical composition of DKP.

Date kernel by-products were first collected and dried at 100 °C, then ground using a grinder to produce a No. 200 sieve size. The properties of DKP are given in Table 1, and its chemical composition is given in Figure 2.

**Figure 3:**
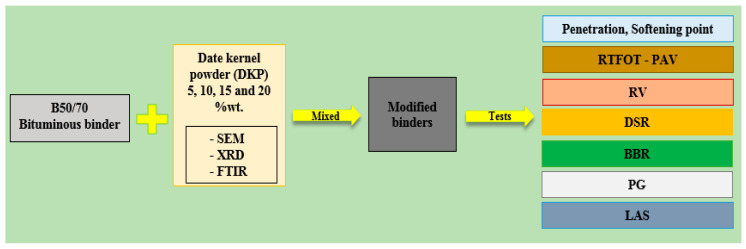
Experimental procedure flow diagram.

Before preparing the modified binders, B50/70 bituminous binder was first heated to approximately 155 °C and liquefied. Then, modified binders were prepared by adding DKP to this bituminous binder at ratios of 5%, 10%, 15%, and 20% by weight. The mixing process was carried out using a high-shear mixer, and the mixing process was performed at a constant temperature of 160 °C for 90 min at a speed of 1900 rpm. The coding of the prepared samples is given in Table 2, and the flow chart of the experiments to be performed on the samples is given in Figure 3.

## 3. Experimental Program

### 3.1. Characterization of DKP

To investigate the micromorphological, crystallographic, and chemical properties of DKP, Scanning Electron Microscopy (SEM), X-ray Diffraction (XRD), and Fourier Transform Infrared Spectroscopy (FTIR) analyses were performed at the Scientific Research Laboratory of İnönü University. SEM analysis examined the surface morphology, porosity, and particle shape of DKP particles; XRD analysis evaluated the crystalline and amorphous phase distribution of the material. FTIR analysis was applied to identify the functional groups present in DKP and to reveal the chemical structure of the material. These analyses aimed to determine the fundamental structural and chemical properties that affect the applicability of DKP in binder modification [47,48].

### 3.2. Traditional Evaluation of Modified Binders

Throughout the study, penetration and softening tests were performed on all original and aged binders. The tests were conducted in accordance with ASTM D5 and ASTM D36 standards, respectively. Additionally, the short-term aging of all binders was achieved using the rolling thin film oven (RTFOT) test according to ASTM D2872, while the long-term aging was achieved using pressurize aging vessel (PAV) tests according to ASTM D6521 standard. Furthermore, the temperature sensitivity of the binders was also calculated using the penetration index (PI) value [21].

### 3.3. RV Tests

The temperatures that binders must maintain during mixing at the plant and during paving on the road are determined using a rotational viscometer (RV) test. The test is performed according to ASTM D4402 standard, where the viscosity–temperature graph of the binders is first plotted. Then, the mixing and compaction temperature ranges (170 ± 20 and 280 ± 30 cP) are determined from this graph, and the average mixing–compaction temperatures are determined from this. The Asphalt Institute recommends that the RV test be performed at temperatures of 135 °C and 165 °C and that the viscosity value at 135 °C not exceed 3000 cP [21].

### 3.4. DSR Test

The DSR test is performed to determine the properties of bituminous binders at high temperatures. The test is performed according to the ASTM D7175 standard, yielding two important parameters: complex shear modulus (G*) and phase angle (δ).

G*, the complex shear modulus, represents the binder’s ability to resist deformation caused by shear stresses, while δ, the phase angle, symbolizes the total resistance ratio in the binder resulting from viscous and elastic responses. Using these two parameters, the rutting parameter (G*/sin δ) and the fatigue parameter (G*·sin δ) are calculated. According to the Superpave specification (AASHTO M320), the rutting parameter is required to be a minimum of 1.0 kPa for unaged binders and 2.2 kPa for RTFOT-aged binders. The fatigue parameter (G*·sinδ) is required to be a maximum of 5000 kPa for PAV-aged binders [1].

### 3.5. BBR Test

The BBR test is applied to determine temperature cracks formed in binders at low temperatures. The test was performed according to ASTM D6648 and the Superpave specification (AASHTO T313). At the end of the test, the creep stiffness (S) and creep rate (m-value) of the binders were determined from the load and deflection values. S represents the resistance to constant creep loads, while the m-value is the ratio between stiffness and loading time. According to the specification, the S value must be max 300 kPa, and the m-value must be min. 0.300 [47].

### 3.6. Determination of the PG

The high and low-temperature performance grades of bituminous binders are indicated by PG X-Y. Here, X represents the high-temperature performance grade, and Y represents the low-temperature performance grade [47].

In this study, the high-temperature performance ratings of the binders were tested at four different temperatures, starting from 52 °C and up to 70 °C. The temperatures in the experiment increased by 6 °C. For performance at intermediate temperatures, 4 different temperatures were used: 16 °C, 19 °C, 22 °C, and 25 °C. For low temperatures, tests were performed at four different temperatures: −16 °C, −22 °C, −28 °C, and −34 °C.

### 3.7. LAS Test

The LAS test is performed to determine the fatigue life of asphalt binders at intermediate temperatures. The test is performed using a DSR testing machine and is conducted according to the AASHTO TP101 standard. The fatigue life of the binders was calculated using Equation (1) based on the Viscoelastic Continuum Damage (VECD) theory, allowing prediction of binder behavior under different deformation levels.

Nf = *A* × (*γp*) − *B* (1)

Here, parameters *A* and *B* represent the coefficients of the VECD theory, Nf represents the fatigue life, and *γp* represents the applied deformation amplitude. The experiments were conducted at a constant temperature of 25 °C [49,50,51].

## 4. Results and Discussion

### 4.1. Chemical and Microstructural Characterization of DKP

**Figure 4:**
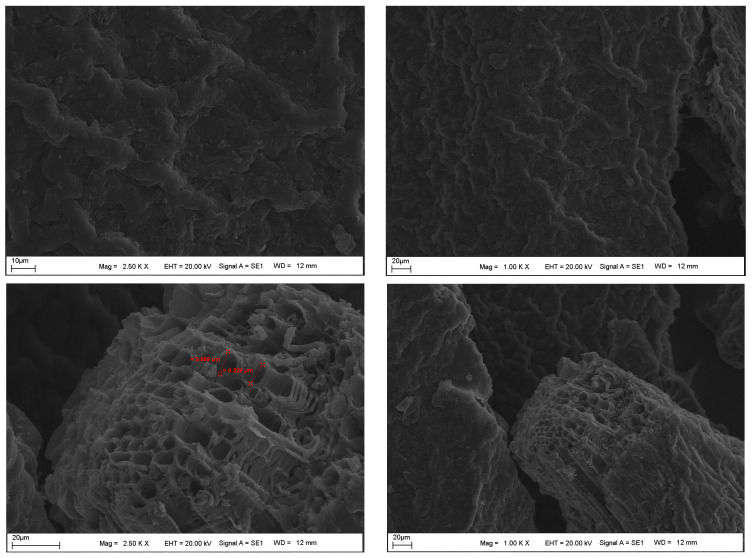
SEM images of DKP.

**Figure 5:**
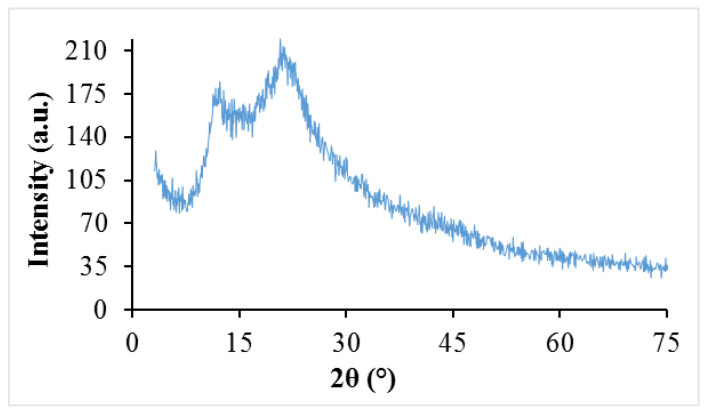
XRD patterns of DKP.

**Figure 6:**
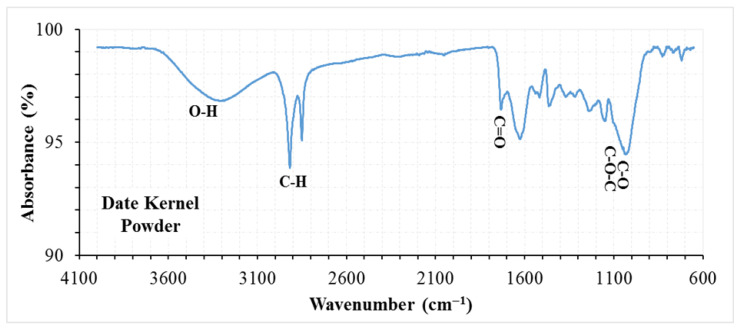
FTIR spectrum of DKP.

The SEM, XRD, and FTIR analysis results for the DKP additive are presented in Figure 4, Figure 5 and Figure 6, respectively.

SEM images of DKP reveal irregular, angular, and brittle particles with rough, heterogeneous surfaces. Micro-cracks, protrusions, depressions, and fibrous structures originating from the lignocellulosic biomass are observed. The irregular pores, measuring a few micrometers, suggest a high specific surface area, which can enhance physical interaction and mechanical interlocking with the bituminous binder matrix.

The XRD pattern of DKP exhibits broad and low-intensity peaks at approximately 2θ ≈ 16° and 2θ ≈ 23°, indicating a predominantly amorphous structure with limited cellulose crystallinity. The broad diffraction features and absence of sharp mineral peaks suggest low inorganic content and a heterogeneous structure dominated by organic components. These findings indicate that DKP behaves as an amorphous biomass material, which may enhance physical interactions in bituminous binder modification.

FTIR spectrum of DKP displays characteristic functional groups of lignocellulosic biomass. The broad band at ~3400 cm^−1^ corresponds to O–H stretching from cellulose and lignin, while bands at 2920–2850 cm^−1^ represent aliphatic C–H stretching. The peak around 1730 cm^−1^ indicates C=O groups from hemicellulose, and the 1600–1510 cm^−1^ bands correspond to aromatic C=C vibrations of lignin. Bands at 1030–1050 cm^−1^ are attributed to C–O and C–O–C vibrations in polysaccharides. No new functional groups are observed, confirming that DKP retains its structural properties during processing.

The microstructural and chemical characteristics observed in SEM, XRD, and FTIR analyses help explain the rheological and performance behavior of DKP-modified binders. The rough and porous morphology observed in SEM images is consistent with the increased viscosity and stiffness measured in RV and DSR tests, suggesting enhanced physical interaction and mechanical interlocking within the bituminous binder matrix. Similarly, the predominantly amorphous structure identified in XRD analysis may contribute to the improved stress relaxation behavior observed in BBR results. FTIR analysis confirmed that no new functional groups were formed, indicating that DKP modification is mainly physical in nature. The consistency of these observations before and after aging suggests that the structural characteristics of DKP remain stable and support the observed rheological performance.

### 4.2. Traditional Test Results

**Figure 7:**
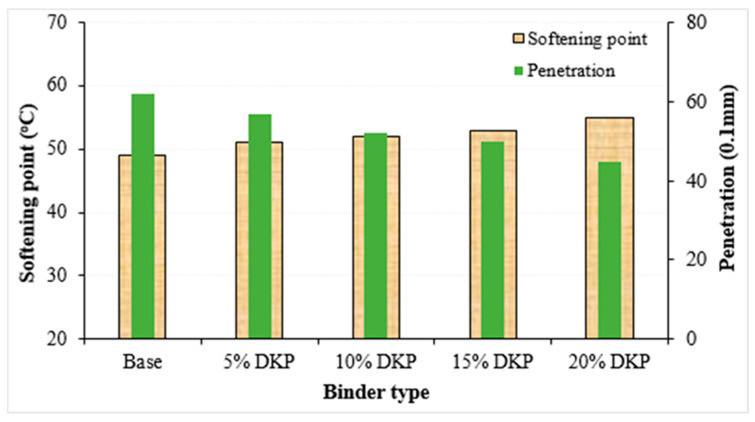
Penetration-softening point values of binders.

In this study, the change in the penetration and softening point values of binders due to DKP contribution is shown in Figure 7, and the changes in PI values and short-term aging properties are shown in Table 3.

It was observed that adding DKP to B50/70 bituminous binder generally increased the softening point value of modified binders and decreased the penetration value. The softening point is an indicator of phase changes in the bituminous binder structure due to temperature. Changes in the softening point are consistent with penetration. Looking at the results, the gradual decrease in penetration values is evidence that modified bituminous binders are physically harder. From this, it is possible to say that DKP-modified binders can be used in coatings in regions with higher temperatures.

When the PI values are examined (Table 3), it is seen that the temperature sensitivity of the binders decreases with the addition of DKP, meaning that the PI values increase.

Looking at the mass losses of the binders determined according to ASTM D2872, it is seen that mass losses gradually decrease with the increase in additive, but remain within the specification limits (max. 0.5). However, improvements are seen in the changes in retained penetration and softening point values with the increase in additive.

Overall, physical tests indicate that DKP increases binder hardness and decreases temperature sensitivity. Considering that the use of DKP additive in bituminous binder modification may be effective on the physical interaction and mechanical interlocking mechanisms of bituminous binder, these results will be directly effective in improving temperature performance.

Similar trends have been reported in previous studies on biomass-based modifiers. Shell-derived and biochar-based additives generally lead to decreases in penetration and increases in softening point due to their rigid lignocellulosic structure and high surface area, which enhance binder–additive interaction [52,53,54,55]. The magnitude of change observed in the present study is comparable to values reported in the literature, although variations between studies may result from differences in base binder grade, additive particle size, and mixing conditions.

### 4.3. RV Results

**Figure 8:**
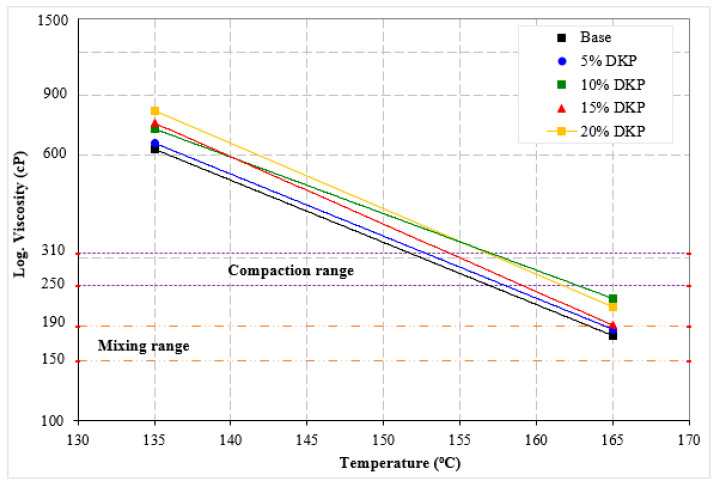
Viscosity–temperature curves.

**Figure 9:**
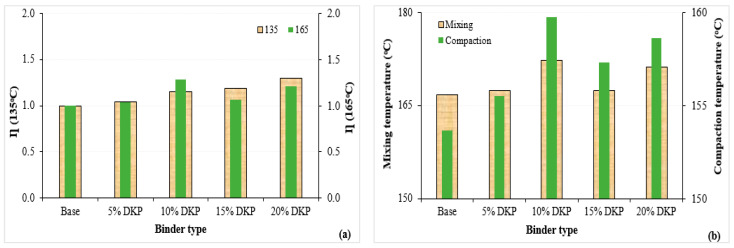
Modification index (**a**) and average mixing–compaction temperatures (**b**) of the binders.

The viscosity–temperature graph obtained from the RV test conducted at 135 °C and 165 °C for the binders is shown in Figure 8. Additionally, the modification index (η) values obtained by dividing the viscosity values of DKP-modified binders by the viscosity of the base bituminous binder were calculated and presented in Figure 9 along with the average mixing and compaction temperatures.

Looking at the viscosity values, it can be seen that, in general, there are increases in viscosity values compared to the base bituminous binder with the additive at both temperature values. These increases are 4.2%, 14.7%, 19.3%, and 30.0% at 135 °C, and 4.4%, 28.5%, 7.2%, and 21.3% at 165 °C, respectively. This result is consistent with the penetration values and indicates an increase in the hardness of DKP-modified binders.

When examining Figure 9, it can be seen that with the increase in DKP, there were increases of 0.42%, 3.4%, 0.48%, and 2.7% in the mixing temperature and 1.2%, 4.0%, 2.4%, and 3.3% in the compaction temperature of the binders compared to the base bituminous binder. The increases in mixing and compaction temperatures are an expected result due to the increased hardness of the modified binders, indicating that binders containing DKP will require increased energy consumption during mixing at the plant and compaction during field application. However, since DKP additive is a residue material and its use in bituminous binder modification will prevent waste, this increase can be considered acceptable in practical applications.

From a practical engineering perspective, the increase in viscosity with DKP addition indicates that slightly higher mixing and compaction temperatures may be required compared to the base binder. Nevertheless, the viscosity values of DKP-modified binders remain within commonly accepted workability limits for asphalt binders. In general practice, a viscosity of approximately 3000 cP at 135 °C is considered an upper practical limit for mixing and handling of asphalt binders. The results suggest that DKP contents up to 15% provide improved performance while maintaining acceptable workability, whereas higher DKP contents may increase production temperatures and energy demand. Therefore, moderate DKP contents appear to provide a reasonable balance between workability and performance improvement.

Comparable viscosity increases have been reported for various biomass-based modifiers, particularly shell-derived and biochar additives, which tend to increase binder stiffness and internal friction due to their rigid particle structure and high surface area [52,53,54,55]. The magnitude of viscosity increase observed in the present study falls within the range typically reported for biomass modifiers. Differences between studies can be attributed to variations in additive dosage, particle size distribution, mixing conditions, and base binder characteristics.

### 4.4. DSR Results

**Figure 10:**
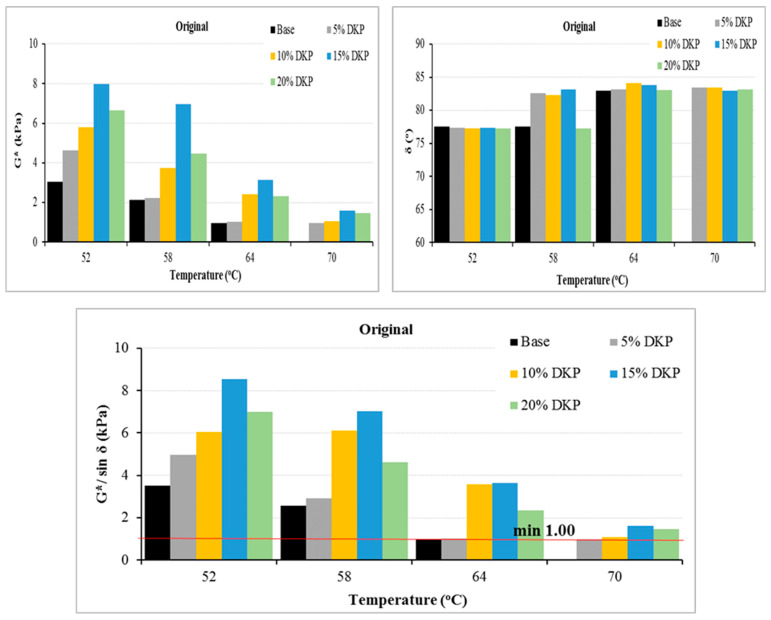
DSR results for the original binders.

**Figure 11:**
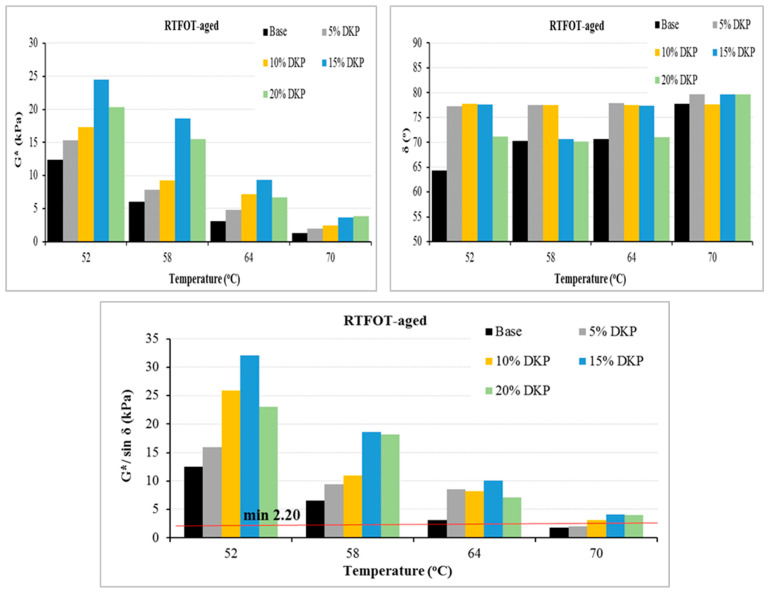
DSR results for RTFOT-aged binders.

**Figure 12:**
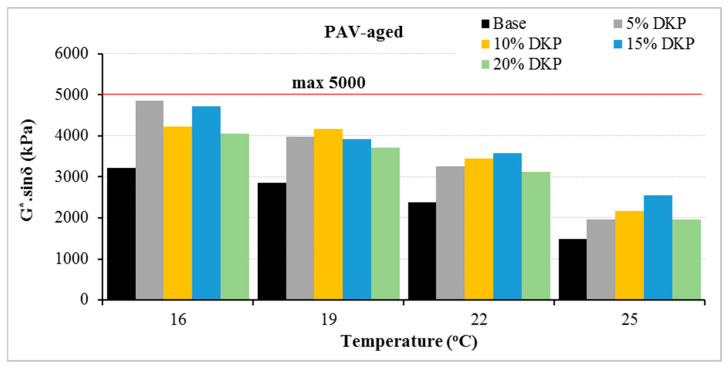
Fatigue parameter results for binders.

The temperature-dependent changes in the rheological parameters of the unaged (original) and RTFOT-aged base and DKP-modified binders are shown in Figure 10, Figure 11 and Figure 12.

Looking at Figure 10 and Figure 11, it can be seen that despite the increase in temperature, the G* values of DKP-modified binders increased compared to the base binder. These increases indicate that DKP increases the total resistance to shear stresses. Looking at the δ values, it can be seen that there were limited increases compared to the base binder. This situation indicates that the addition of DKP partially increases the viscous component of the binder; however, when evaluated together with the significant increase in G* values, it shows that the modified binder exhibits more resistant and balanced rheological behavior against shear stresses at high temperatures.

When examining the G*/sin δ parameters, it is seen that DKP-modified binders exhibit a significant increase in rutting resistance compared to the base binder. The increase in G*/sin δ values indicates that DKP modification effectively increases the elastic contribution of the binder, limiting viscous flow and suppressing permanent shape changes under load. This confirms that DKP-modified binders offer superior rutting resistance compared to base binders at service temperatures.

When evaluating the high-temperature performance levels of binders according to the Superpave specification, the base binder was determined to be in the PG 64-Y level. With the increase in the DKP additive ratio, the performance levels of the modified binders were found to change to PG 64-Y, PG 70-Y, PG 70-Y, and PG 70-Y, respectively. This indicates that the addition of DKP increases the rigidity and resistance to deformation of the binder at high temperatures, thereby raising the high-temperature performance grade by one level. The fact that the PG level remains constant in modified binders suggests that the additive’s effectiveness on the binder has reached saturation and that further increases in DKP have a limited effect on high-temperature performance. Therefore, it can be said that DKP-modified binders offer advantages in the design of asphalt mixtures that can be used in hot climates and under heavy traffic loads.

The G*·sinδ results obtained after PAV aging show that DKP-modified binders meet the 5000 kPa limit specified in the Superpave specification at all temperature levels. This demonstrates that DKP modification increases the stiffness of the binder while maintaining the necessary deformation capacity against fatigue cracking. The regular decrease in G*·sinδ values observed with increasing temperature indicates that the binders exhibit more ductile behavior, reducing their tendency to initiate cracks under repeated loads. In general, it can be stated that the addition of DKP provides a balanced rheological behavior between stiffness and flexibility due to the structural ordering it creates within the binder matrix and maintains acceptable fatigue performance under long-term aging conditions.

Overall, DSR results indicate that DKP modification enhances the binder’s rheological behavior in multiple ways. The increase in complex modulus (G*) and G*/sinδ values shows improved resistance to shear stresses and permanent deformation at high temperatures. This is confirmed by the binder’s upgrade from PG 64-Y to PG 70-Y in the Superpave high-temperature performance classification. On the other hand, the limited changes in phase angle and the fact that the G*·sinδ values obtained after PAV aging remain within the specification limits indicate that the DKP modification maintains the binder’s necessary deformation capacity against fatigue cracking despite the increased stiffness. In conclusion, the DKP additive significantly improves the binder’s high-temperature performance without adversely affecting its fatigue behavior; it provides a balanced rheological structure between stiffness and flexibility, offering a suitable modification alternative for hot climates and heavy traffic conditions.

Similar improvements in rheological properties and rutting resistance have been reported for various biomass-based modifiers, particularly shell-derived and biochar additives, which commonly increase complex modulus and rutting parameters while maintaining acceptable viscoelastic balance [52,53,54,55]. The improvement in high-temperature performance grade observed in this study (PG 64 to PG 70) is consistent with the PG shifts typically reported for lignocellulosic biomass modifiers. Differences in the magnitude of rheological improvement reported in the literature may be associated with variations in base binder grade, additive processing and grinding conditions, modifier dosage, mixing protocol, and aging procedures.

### 4.5. BBR Results

**Figure 13:**
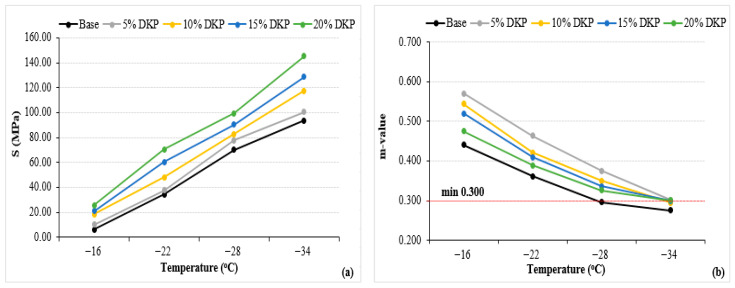
S (**a**) and m-values (**b**) for binders.

The creep stiffness (S) and creep rate (m-value) values of the binders are given in Figure 13.

When the obtained values are examined, it is seen that the addition of DKP increases both the S and m-value of the binders. It can be understood from the figure that the creep values increase as the temperature decreases but still remain within the specification limit (max. 300 kPa). This indicates that the binders behave more like an elastic solid. Looking at the m-value results, it is seen that the values generally decrease with increasing temperature and, especially after −28 °C, the binders with additives (except for 15% and 20% DKP) cannot meet the specification limit (min. 0.300). These results show that DKP-modified binders are not significantly affected by low temperatures despite their hardening.

When S and m-value are evaluated together, the low-temperature performance grade of the base binder is PG X-22, while the DKP-modified binders, with increasing modification ratios, are determined to be PG X-28, PG X-28, PG X-34, and PG X-34, respectively. The results show that DKP-modified binders are more resistant to cracking at low temperatures. This behavior is thought to be related to the lignocellulosic structure and porous morphology of DKP, which may contribute to improved stress relaxation capacity at low temperatures.

Comparable improvements in low-temperature performance have been reported for various biomass-based modifiers, where lignocellulosic particles contribute to improved stress relaxation and crack resistance despite increased binder stiffness [52,53,54,55]. The improvement in low-temperature performance grade observed in this study is consistent with trends reported for shell-derived and biochar modifiers. Variations reported in the literature are generally attributed to differences in modifier content, particle size, binder source, and testing temperature range.

### 4.6. PG Values

The high- and low-temperature performance levels of the base and DKP-modified binders are given in Table 4 below, while true high- and low-temperature values are given in Table 5.

Table 4 shows that the addition of DKP improves the performance of modified binders in both high- and low-temperature classes. In particular, at a 15% DKP ratio, both temperature levels show an increase compared to other binders, which is a critical point. Ultimately, it is possible to say that binders with 15% DKP content can be quite resistant to rutting mark formation at high temperatures and thermal cracking at low temperatures. This performance increase can be attributed to DKP’s lignocellulosic structure and the polar functional groups it contains, which are believed to enhance the internal structure of the binder matrix, thereby contributing to improved performance at both high and low temperatures.

The true performance grade temperatures presented in Table 5 provide a more precise evaluation of binder performance compared to the standard PG classification given in Table 4. The true high-temperature grades increase progressively with DKP content, indicating improved resistance to permanent deformation. The true high-temperature value of the base binder was determined as approximately 66.7 °C, while the modified binders reached values exceeding 70 °C.

The true low-temperature grades also indicate improved resistance to thermal cracking with DKP addition. The base binder exhibited a true low-temperature value of approximately −27.6 °C, whereas DKP-modified binders reached true low-temperature values approaching −34 °C. These results are consistent with the PG classification presented in Table 4 and confirm that DKP modification improves binder performance over a wider temperature range.

As a biomass-based material, date kernel powder contains organic components that may be more sensitive to environmental aging factors such as moisture, oxygen, and ultraviolet radiation compared with inorganic mineral fillers [32]. In the present study, the aging characteristics of DKP-modified binders were evaluated through RTFOT and PAV procedures representing short- and long-term oxidative aging conditions, and the results indicate that the modified binders maintain satisfactory rheological and performance properties after aging. These findings suggest that DKP-modified binders can provide stable performance under oxidative aging conditions. Further studies involving combined environmental effects such as moisture conditioning and UV exposure would provide a more comprehensive understanding of the long-term durability of DKP-modified binders.

Similar improvements in temperature performance range and aging resistance have been reported for lignocellulosic biomass modifiers, where the interaction between biomass particles and the binder matrix contributes to improved thermal cracking resistance and stable rheological properties after aging [52,53,54,55]. Differences reported in the literature are generally associated with variations in base binder characteristics, additive preparation methods, and aging procedures.

### 4.7. LAS Results

**Figure 14:**
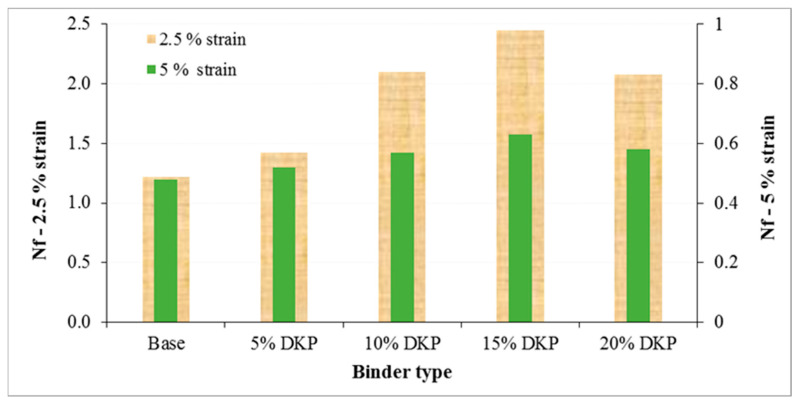
LAS test results of the binders.

Unlike the DSR fatigue parameter, the LAS test provides a more realistic assessment of binder fatigue behavior under increasing strain amplitudes, allowing the evaluation of damage evolution and fatigue life prediction. In addition to the DSR test, the fatigue life of base and DKP-modified binders was evaluated using the LAS test. The fatigue life (Nf) of all binders is shown in Figure 14.

The LAS test results show that the DKP additive has a positive effect on the fatigue performance of the binders. Examining the fatigue life (Nf) values presented in Figure 14, it is seen that DKP-modified binders achieve a higher number of cycles compared to the base binder at both 2.5% and 5% strain levels. The more pronounced increase in fatigue life at the 2.5% strain level, in particular, indicates that the DKP additive increases the crack initiation resistance of the binder under low and intermediate strain conditions.

The steady increase in fatigue life up to a 15% DKP level as the DKP ratio increases suggests that the additive creates a more balanced viscoelastic structure between stiffness and deformation capacity within the binder matrix. The limited decrease in fatigue life at a 20% DKP ratio indicates that at high additive ratios, the rigidity effect may become dominant, partially limiting the deformation capacity.

The results obtained at a 5% strain level reveal that DKP-modified binders maintain their fatigue performance even under high deformation conditions and offer a longer fatigue life compared to base binders. These findings are consistent with the fatigue parameter (G*·sinδ) results obtained from DSR tests, showing that DKP modification increases the stiffness of the binder without causing excessive brittleness.

In conclusion, it can be stated that DKP addition provides a balanced performance between stiffness and flexibility without adversely affecting the fatigue behavior of the binder, and that optimum effectiveness is achieved at a 15% DKP ratio.

Similar fatigue performance trends have been reported for various biomass-based modifiers, where moderate additive contents improve fatigue resistance by enhancing the viscoelastic balance of the binder without causing excessive brittleness [52,53,54,55]. The fatigue behavior observed in the present study is consistent with these findings. Variations reported in the literature are generally attributed to differences in modifier dosage, particle dispersion, binder type, and testing strain levels.

### 4.8. Comparison with Other Biomass Modifiers

To better understand the magnitude of improvement provided by DKP, the rheological and performance results obtained in this study were compared with those reported for similar biomass-based asphalt binder modifiers in the literature.

The addition of DKP resulted in a noticeable improvement in the rheological performance of the binders. In particular, the high-temperature performance grade increased from PG 64 for the base binder to PG 70 for the DKP-modified binders, indicating an improvement of one PG grade (approximately 6 °C). In addition, DKP modification led to an increase in viscosity and rutting resistance parameters, demonstrating enhanced resistance to permanent deformation at high temperatures.

Similar improvements have been reported for other lignocellulosic biomass modifiers. For example, hazelnut shell biochar has been reported to increase binder viscosity and improve rutting resistance, indicating enhanced high-temperature performance. The improvement obtained with DKP in this study is comparable to the performance enhancement reported for hazelnut shell biochar-modified binders, suggesting that DKP can provide similar benefits to other shell-based biomass modifiers [52].

Biochar-based modifiers have also been widely studied as sustainable asphalt binder additives. Previous studies have shown that biomass-derived biochar modifiers can improve rutting resistance and rheological properties, typically resulting in rutting resistance increases of approximately 10–18%, depending on the additive content. The improvement observed with DKP modification in this study is consistent with these findings, indicating that DKP provides a level of performance enhancement comparable to conventional biochar-based modifiers [53].

Coconut-shell-based modifiers have also been reported to improve the rheological performance of asphalt binders. The addition of coconut shell nanocharcoal ash has been shown to decrease penetration values, increase softening point and viscosity, and improve rutting resistance of bituminous binder. For example, coconut shell nanocharcoal ash modification increased the rutting resistance temperature from approximately 64 °C to about 70 °C and increased viscosity values compared with the base binder. Similar improvements were observed in the present study, where DKP modification resulted in increased viscosity and enhanced rutting resistance. These results indicate that DKP provides performance improvements comparable to coconut-shell-based biomass modifiers [54].

Previous studies have also indicated that biomass-derived modifiers generally increase binder stiffness and improve rutting resistance due to their lignocellulosic structure and high carbon content. These modifiers typically lead to increases in viscosity and softening point, contributing to improved high-temperature performance. The behavior observed for DKP-modified binders in the present study is consistent with these general trends reported for biomass-based asphalt modifiers [55].

In contrast to biochar and shell-based modifiers, bio-oil modifiers such as cottonseed oil are generally reported to reduce binder stiffness and viscosity while improving workability. Previous studies have shown that the addition of cottonseed oil decreases rutting resistance parameters and binder viscosity. Unlike these bio-oil modifiers, DKP modification in the present study resulted in increased viscosity and improved rutting resistance without compromising the overall performance of the binder. This indicates that DKP provides a more balanced improvement in binder performance compared to some bio-based softening additives [56].

Overall, the results demonstrate that DKP performs at least at the same level as commonly used biomass modifiers while offering the additional advantage of simple and direct utilization without combustion or chemical treatment.

## 5. Conclusions

The main results obtained in this study, which investigated the usability of date kernel powder (DKP) in bituminous binder modification, are summarized below.

❖ SEM, XRD, and FTIR analyses indicate that DKP possesses a rough and porous surface, is predominantly amorphous, and contains polar functional groups such as hydroxyl, carbonyl, and aromatic groups. These characteristics suggest that DKP may enhance physical and mechanical interactions within the bituminous binder matrix.
❖ The results of physical tests demonstrate that incorporating DKP increases the hardness of the binders and reduces their temperature sensitivity. This effect is likely due to the lignocellulosic and porous nature of DKP, which strengthens binder–additive interactions and supports mechanical interlocking within the matrix.
❖ According to DSR results, the DKP additive increased the high-temperature performance level of binders by 6 °C, raising it from PG 64-Y level to PG 70-Y level at a 15% DKP ratio. The increase in complex modulus suggests that DKP contributes to improved resistance to permanent deformation at high temperatures.
❖ BBR tests indicate that DKP addition improves resistance to thermal cracking and enhances the low-temperature performance grade from PG-22 to PG-34 at a 15% DKP ratio, based on the Superpave grading criteria using creep stiffness (S ≤ 300 kPa) and m-value (m ≥ 0.300). This improvement may be associated with the amorphous-dominant structure and polar functional groups of DKP, which may support stress relaxation capacity at low temperatures.
❖ When evaluating the high and low temperature performance levels of the binders together, it was observed that the performance level of the base binder, which was PG 64-22, increased to PG 70-34 with a 15% DKP additive. This indicates that DKP-modified binders show improved resistance to rutting deformation at high temperatures and thermal cracking at low temperatures.
❖ LAS test results show that DKP-modified binders exhibit similar or higher fatigue life than the base binder at both 2.5% and 5% strain levels. While fatigue life increased with DKP content up to 15%, the limited decrease observed at a 20% DKP ratio indicates that increased rigidity at high additive levels may partially limit deformation capacity. Overall, the LAS findings confirm that DKP modification increases fatigue resistance without causing excessive brittleness and provides balanced viscoelastic behavior at moderate temperatures.

In conclusion, the use of date kernel powder, an agricultural by-product, in bituminous binder modification improves the rheological and performance properties of binders at the binder level. DKP addition improves resistance to permanent deformation at high temperatures while also improving thermal cracking resistance and fatigue behavior at intermediate temperatures. A DKP content of 15% appears to provide the most balanced performance considering performance grade improvement, fatigue behavior, and workability characteristics, while higher contents may lead to excessive stiffness. In this context, DKP can be considered a promising modifier for bituminous binders. However, further studies at the mixture and field scale are recommended to confirm the long-term engineering applicability of DKP-modified binders.

## Figures and Tables

**Table 1:** Properties of DKP.

Properties	DKP
Odor	Odorless
Form	Powder
Color	Yellowish
Water solubility	Insoluble
Structure	Fibrous

**Table 2:** Coding of binders.

Sample	Coded
B 50/70 bituminous binder	Base
B 50/70 bituminous binder + 5% DKP	5% DKP
B 50/70 bituminous binder + 10% DKP	10% DKP
B 50/70 bituminous binder + 15% DKP	15% DKP
B 50/70 bituminous binder + 20% DKP	20% DKP

**Table 3:** PI values and short-term aging characteristics of the binders.

Binder Type	PI	Mass Loss (%)	Softening Point Change (°C)	Retained Penetration (%)
Base	−0.95	0.32	5.00	85.00
5% DKP	−0.65	0.27	4.50	86.40
10% DKP	−0.63	0.25	4.50	86.10
15% DKP	−0.48	0.21	4.00	87.20
20% DKP	−0.28	0.13	3.50	87.00

**Table 4:** High- and low-temperature performance grades of binders.

Binder Type	High-Temperature Performance Grade	Low-Temperature Performance Grade	PG X-Y
Base	PG 64-Y	PG X-22	PG 64-22
5% DKP	PG 64-Y	PG X-28	PG 64-28
10% DKP	PG 70-Y	PG X-28	PG 70-28
15% DKP	PG 70-Y	PG X-34	PG 70-34
20% DKP	PG 70-Y	PG X-34	PG 70-34

**Table 5:** The true high- and low-temperature values of the binders.

Binder Type	True High Temperature (°C)	True Low Temperature (°C)
Base	66.70	−27.64
5% DKP	69.40	−33.16
10% DKP	71.30	−33.45
15% DKP	71.60	−34.00
20% DKP	72.20	−34.24

## Data Availability

The original contributions presented in this study are included in the article. Further inquiries can be directed to the corresponding author.
